# Multivariate decoding of brain images using ordinal regression^☆^

**DOI:** 10.1016/j.neuroimage.2013.05.036

**Published:** 2013-11-01

**Authors:** O.M. Doyle, J. Ashburner, F.O. Zelaya, S.C.R. Williams, M.A. Mehta, A.F. Marquand

**Affiliations:** a King's College London, Department of Neuroimaging, Institute of Psychiatry (PO89), De Crespigny Park, London SE5 8AF, UK; b Wellcome Trust Centre for Neuroimaging, 12 Queen Square, London WC1N 3BG, UK

**Keywords:** Multivariate, Ordinal regression, Gaussian processes, Pharmacological MRI, Ketamine, Scopolamine

## Abstract

Neuroimaging data are increasingly being used to predict potential outcomes or groupings, such as clinical severity, drug dose response, and transitional illness states. In these examples, the variable (target) we want to predict is ordinal in nature. Conventional classification schemes assume that the targets are nominal and hence ignore their ranked nature, whereas parametric and/or non-parametric regression models enforce a metric notion of distance between classes. Here, we propose a novel, alternative multivariate approach that overcomes these limitations — whole brain probabilistic ordinal regression using a Gaussian process framework. We applied this technique to two data sets of pharmacological neuroimaging data from healthy volunteers. The first study was designed to investigate the effect of ketamine on brain activity and its subsequent modulation with two compounds — lamotrigine and risperidone. The second study investigates the effect of scopolamine on cerebral blood flow and its modulation using donepezil. We compared ordinal regression to multi-class classification schemes and metric regression. Considering the modulation of ketamine with lamotrigine, we found that ordinal regression significantly outperformed multi-class classification and metric regression in terms of accuracy and mean absolute error. However, for risperidone ordinal regression significantly outperformed metric regression but performed similarly to multi-class classification both in terms of accuracy and mean absolute error. For the scopolamine data set, ordinal regression was found to outperform both multi-class and metric regression techniques considering the regional cerebral blood flow in the anterior cingulate cortex. Ordinal regression was thus the only method that performed well in all cases. Our results indicate the potential of an ordinal regression approach for neuroimaging data while providing a fully probabilistic framework with elegant approaches for model selection.

## Highlights

• Often in neuroimaging the independent variables are ranked or ordered.
• Classification and regression models cannot explicitly model an ordinal target.
• We present a novel multivariate ordinal regression approach for neuroimaging data.
• Our results show that ordinal regression is a powerful method for ranking data.

## Introduction

Neuroimaging can be used to investigate a wide range of clinical and research questions including disease processes (Fusar-Poli et al., in press; Jack et al., 2008) and decode both instantaneous cognitive states (Friston et al., 2008) and pharmacological intervention (Deakin et al., 2008). Increasingly, multivariate pattern recognition techniques are being applied to neuroimaging data to answer fundamental questions based around diagnosis/prognosis and decoding. Primarily, this can be achieved by training a learning machine using a subset of the data and their respective targets (e.g. clinical measure or state label) and observing the accuracy of the target assigned to a set of ‘unseen’ data, which serves to estimate the generalisation performance of the learner. Most commonly, learning machines are used to perform binary classification whereby only two labels or states are considered and the classes are assumed to have a nominal relationship to one another (e.g. patient vs. control or placebo vs. drug). To date, the most popular approach has been the binary support vector machine classifier (Fan et al., 2007; Mourao-Miranda et al., 2012; Pantazatos et al., 2012; Plant et al., 2010).

In order to consider more than two labels, multi-class learning can be employed. To perform multi-class classification a common approach, in the field of neuroimaging, has been to split the problem up into a series of binary classification problems and then apply combination strategies such as error correcting codes (Hassabis et al., 2009; Mourao-Miranda et al., 2006; Schrouff et al., 2012) or “one-versus-all” (Chu et al., 2011; Zheng et al., 2013). Alternatively, several studies have focused on the use of a single learning machine in order to consider all classes in a mutual context. Marquand et al. (2012) applied sparse multinomial logistic regression to pharmacological imaging data in order to discriminate cerebral blood flow maps collected using arterial spin labelling after placebo, atomoxetine and methylphenidate administration. Filippone et al. (2013) used an inherently multi-class Gaussian process classification approach for neuroimaging data to discriminate between different Parkinsonian neurological disorders and healthy controls. Jenatton et al. (2012) investigated the performance of several sparse methods using both an inherently multi-class likelihood and a “one-versus-all” approach to discriminate the mental representation of different objects. The authors conclude that the best performance was achieved using a multinomial likelihood within their framework.

For many applications of neuroimaging, the class labels can be ordered or ranked, but it can be difficult to exactly quantify the distance between the categories. One example is a disease process continuum where the labels of scans can be ordered as: healthy controls, prodromal and disease state. The most cited example in the neuroimaging literature being the Alzheimer's Disease Neuroimaging Initiative (ADNI) data set which contains neuroimaging and clinical data from participants with Alzheimer's disease, progressive mild cognitive impairment, stable mild cognitive impairment and those who are cognitively normal (Mueller et al., 2005). Similarly, there is a lot of interest in identifying and monitoring individuals at risk of psychosis, who may be in a prodromal phase of the disease (Fusar-Poli et al., in press). For example, Borgwardt et al. (in press) analysed structural MRI scans from a psychosis continuum (healthy controls–at risk–first-episode psychosis) albeit using binary pairs of classifiers.

In addition to disease trajectories, continuum models are also evident in several other applications of neuroimaging. Increasing complexity or difficulty of cognitive tasks can be framed as a continuum; even if the task complexity can be accurately quantified (e.g. linear increase in the number of items to be remembered), the resulting increase in neural processing should not be assumed to follow the same pattern. Similarly, dose response relationships with neuroimaging data represent a continuum where the known dose interval may not match the differences in brain states (Tauscher et al., 2002). This may be particularly apparent when a small number of doses are used, or a new mechanism is being investigated. In all these examples, it would seem desirable to make use of the ordinal relationships between class labels to enable them to be more accurately predicted, compared to binary or multi-class classification approaches which ignore this information.

Here, we pose the question: how can we identify a state that is assumed to be intermediate between multiple states? We propose re-formulating this problem using a multivariate ordinal regression framework which inherently models the natural ordering in the data labels and can establish whether a collection of brain regions are ordinally related across a continuum. Crucially, this framework will consider all classes simultaneously and can be tested on a single test case (we do not require a test instance for each class to decode the ranking) in contrast to the only two previous ordinal ranking approaches for neuroimaging of which we are aware; Fan and ADNI (2011) decomposed the problem into pairwise classifiers which were then combined using an ordinal ranking rule while Pedregrosa et al. (2012) proposed training a single binary classifier to discriminate pairs of data vectors that exhaustively describe all pairs of classes. This method requires that a test case for each class must be available to decode the ranking of the image. We focus in particular on ordinal regression as conventional parametric or non-parametric regression approaches enforce a metric notion or sense of symmetry between labels. Consider the ordinal scale mild, medium and severe. To use a regression approach we may encode these labels as [1, 2, 3]. However, this metric notion assumes that the difference between mild and medium is the same as the difference between medium and severe. Additionally, unlike multi-class classification, which provides a different set of predictive weights for each class the model structure for an ordered class relation typically involves estimating a single set of predictive weights which reflects the ordering of all the classes (Chu and Ghahramani, 2005; Gutiérrez et al., 2012; Mccullagh, 1980). Moreover, performance metrics should be appropriately chosen as for ordinal regression the ‘distance’ of the error from its true label is of interest. That is, the magnitude of the error on incorrectly classifying mild as severe should be more highly penalised than classifying mild as moderate.

To investigate whether multivariate ordinal regression is a better suited approach for data with ordered targets we utilise two exemplar pharmacological imaging studies. The first study aims to investigate the blood oxygen level-dependent (BOLD) response to ketamine which acts as an N-methyl D-aspartate (NMDA) receptor antagonist and evokes psychotomimetic symptoms resembling schizophrenia in healthy humans (Krystal et al., 1994). Imaging markers of acute ketamine challenge have the potential to provide a powerful assay of novel therapies for psychiatric illness. In this data set, the modulation of the BOLD response to ketamine is investigated using two compounds — the anticonvulsant lamotrigine and the antipsychotic risperidone (Doyle et al., 2013). In our earlier paper, we confirmed that both lamotrigine and risperidone attenuate the effects of ketamine on the BOLD phMRI signal, albeit via different mechanisms of action (Doyle et al., 2013). We thus expect the class labels to be ordinal with placebo and ketamine at the extremities and lamotrigine or risperidone as the intermediate class. As this study was carried out to explore the level of attenuation achieved by risperidone and compare and replicate the attenuation of ketamine by lamotrigine, a priori we cannot rank lamotrigine and risperidone in a meaningful, principled manner, hence we will apply the algorithms separately. The second study focuses on the use of scopolamine which has been used for many years as a pharmacological model of ‘cholinergic amnesia’ gaining popularity due to the cholinergic hypothesis of geriatric memory dysfunction (Bartus et al., 1982), and the reversal of deficits is widely adopted as a tool to test putative cognitive-enhancing drugs. This study utilises arterial spin labelling to investigate the effect of scopolamine (a potent antagonist of the muscarinic acetylcholine receptor) on cerebral blood flow and its modulation using donepezil, an acetylcholinesterase inhibitor which can provide some improvement in cognitive impairments in cholinergic animal models of Alzheimer's disease as well as patients (Di Santo et al., 2013; Winblad et al., 2001). Considering that scopolamine is a non-selective muscarinic acetylcholine receptor antagonist whereas donepezil will enhance cholinergic transmission to both nicotinic and muscarinic receptors we do not expect the whole brain response to be ordinal as donepezil will produce specific regional CBF (rCBF) effects distinct from scopolamine. Therefore, we expect donepezil to attenuate scopolamine effects in regions which are rich in muscarinic receptors and have previously shown a response to scopolamine. Hence we use ordinal regression to explore the rCBF in predefined regions of interest (ROIs). Previous neuroimaging studies have shown both increases and decreases in cerebral blood flow following scopolamine administration, although these effects were not derived from fully quantitative measurements of blood flow (Grasby et al., 1995; Honer et al., 1988; Prohovnik et al., 1997). Work in experimental animals has demonstrated reduced CBF following scopolamine (Ogawa et al., 1994; Tota et al., 2012), which can be reversed by acetylcholinesterase inhibitors (Ogawa et al., 1994; Tsukada et al., 1997). Therefore, we expect rCBF to be most reduced following scopolamine administration and we expect rCBF following pre-treatment with donepezil to lie between that of placebo and scopolamine.

In this work, we describe a whole brain probabilistic approach for ordinal regression using Gaussian processes (ORGP) in a Bayesian framework. This framework provides probabilistic predictions for class membership which facilitates the quantification of uncertainty, and an elegant approach for model selection and comparison. The likelihood function of this method specifically captures the ordinal nature of the data using a threshold model which is a generalisation of the probit function as introduced by Chu and Ghahramani (2005). We will compare this approach to schemes for multi-class classification and metric regression, in all cases linear learning algorithms will be utilised. Comparisons will be carried out using three data configurations: 1) BOLD data ranked increasingly as placebo, lamotrigine–ketamine and ketamine, 2) BOLD data ranked increasingly as placebo, risperidone–ketamine and ketamine and 3) ASL data ranked decreasingly as placebo, donepezil–scopolamine and scopolamine. Recalling that the intermediate classes feature the compound of interest (i.e. ketamine or scopolamine) administered in addition to a modulatory pre-treatment (i.e. lamotrigine, risperidone or donepezil), which would be expected to attenuate the effects of the compound of interest.

## Materials and methods

### ORGPs — ordinal regression using Gaussian processes^2^

Here, we provide an insight into the mathematical concepts proposed by Chu and Ghahramani (2005) which govern this approach to ordinal regression. We refer the reader to Chu and Ghahramani (2005) for a more detailed description of this method.

Consider a *training* data set D of *N* observations, $D=\left\{\left(x_{i},y_{i}\right)|i=1,\ldots ,N\right\}$ where each sample is a pair consisting of the input data vector ***x**_i_* of dimension *M* and corresponding label *y*_*i*_ ∈ *L* where *L* is a finite set of *R* ordinal categories, denoted *L* = {1, 2, …*R*}. The column data vectors for all *N* cases are aggregated in the data matrix ***X*** with dimensions *N* × *M* and the targets are collected in vector ***y***.

The main principle here is to assume an unobservable latent function $f\left(x_{i}\right)\in R$ associated with each ***x**_i_* and assume a Gaussian process prior over **f**, where **f** is a vector collecting all latent function values. The ordinal variable *y*_*i*_ is dependent on the latent function *f*(**x**_*i*_) by modelling the ranks as intervals on the real line (Chu and Ghahramani, 2005). This is achieved using a Bayesian framework. First, a Gaussian process prior *P*(**f**|**X**, **θ**) is placed on the latent function. The Gaussian process prior can be fully defined by a mean function *m*(**x**) and a covariance function *k*(**x**_*i*_,**x**_*j*_). Here we define the GP as zero mean with a linear covariance matrix:

$$K=\left(XX^{T}+1\right)/s^{2}$$

where *s*^2^ is a bias term that also controls the scaling of the latent function which in turn affects the variance of the predictive weights. We refer to this quantity as a hyperparameter, collected in the vector **θ**, which will be optimised within this framework.

The joint probability of observing the ordinal variables, i.e. the likelihood is defined as

$$P\left(y|f\right)=\overset{N}{\underset{i=1}{\prod }}P\left(y_{i}|f\left(x_{i}\right)\right)\text{.}$$

**Fig. 1:**
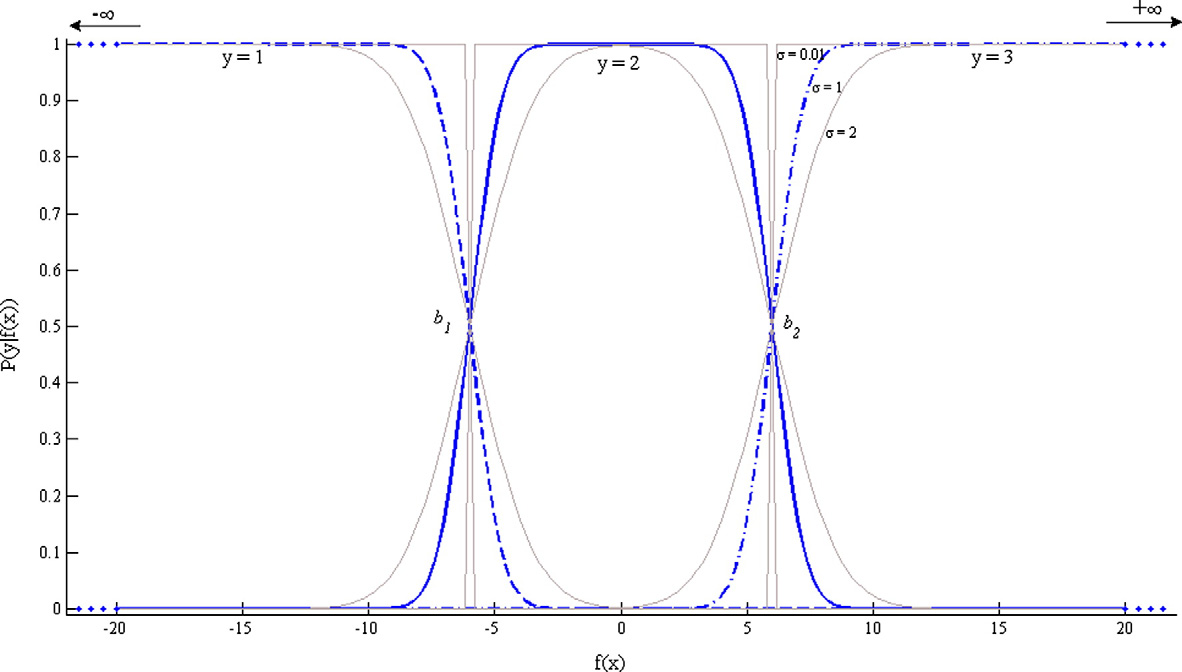
Ordinal regression likelihood functions for a problem with three ordinal classes (*R* = 3) and hence two threshold variables with *b*_1_ = − 6 and *b*_2_ = 6 and two extremity threshold constants *b*_0_ and *b*_3_ which are set to − ∞ and + ∞, respectively. The case for the noise parameter *σ* = 1 appears in boldfaced blue and two additional greyed out functions are displayed for *σ* = 0.01 (approximating the noise-free case) and *σ* = 2.

Under noise-free conditions, the ideal likelihood function would be defined as

$$P_{\mathrm{ideal}}\left(y_{i}|f\left(x_{i}\right)\right)=\left\{\begin{array}{l} 1,\;\mathrm{if}\;b_{y_{i}-1}<f\left(x_{i}\right)\leq b_{y_{i}}\\ 0,\;\mathrm{otherwise} \end{array}\right)$$

where *b* represents a threshold variable. At the extremities these variables serve as limits and are defined as *b*_0_ = − *∞* and *b*_*R*_ = + *∞* and the intermediate thresholds are further defined as *b*_*j*_ = *b*_1_ + ∑_*l* = 2_^*j*^*Δ*_*k*_ with positive padding variables *Δ*_*k*_ where *k* = 2,…,*R* − 1. This formulation enforces ordinal constraints by dividing the real line into *R* contiguous intervals (*b*_1_ < *b*_2_ < … < *b*_*R* − 1_) which map *f*(**x**_*i*_) to the discrete variable *y*_*i*_. Note that the thresholds are not constrained to be equidistant. A schematic illustration of the shape of the likelihood functions for a three class problem is provided in Fig. 1.

*P*_*ideal*_(*y*_*i*_|*f*(**x**_*i*_)) assumes that the input data are noise-free. To account for noise, we explicitly assume that the latent functions are contaminated by Gaussian noise with zero mean and unknown variance *σ*^2^, denoted $N\left(\delta ;0,\sigma^{2}\right)$ where *δ* is a Gaussian random variable. The variance term here controls the shape of the likelihood function serving to sharpen or soften the thresholds (see Fig. 1). The ordinal likelihood function becomes

$$P\left(y_{i}|f\left(x_{i}\right)\right)=\int P_{\mathrm{ideal}}\left(y_{i}|f\left(x_{i}\right)+\delta \right)N\left(\delta ;0,\sigma^{2}\right)\mathrm{d\delta}=\Phi \left(z_{1}^{i}\right)-\Phi \left(z_{2}^{i}\right)$$

where $z_{1}^{i}=\frac{b_{y_{i}}-f\left(x_{i}\right)}{\sigma }$ and $z_{2}^{i}=\frac{b_{y_{i}-1}-f\left(x_{i}\right)}{\sigma }$ and Φ(*z*) is the cumulative unit Gaussian whereby $\Phi \left(z\right)=\int_{-\infty }^{z}N\left(\varphi ;0,1\right)\mathrm{d\varphi}$.

Bayes' theorem can be used to compute the posterior probability, hence enabling predictions to be made.

#### Bayesian framework

From Bayes' theorem the posterior probability distribution over the latent function can be written as

$$P\left(f|y,X,\theta \right)=\frac{1}{P\left(y|X,\theta \right)}\;\overset{N}{\underset{i=1}{\prod }}P\left(y_{i}|f\left(x_{i}\right)\right)P\left(f|X,\theta \right)$$

where the prior probability is defined as $P\left(f|X,\theta \right)={\left(2\pi \right)}^{-\frac{N}{2}}|K{|}^{-\frac{1}{2}}\;\exp\left(-\frac{1}{2}f^{T}K^{-1}f\right)$ where ***K*** is the covariance matrix and *P*(**y**|**X**, **θ**) = ∫*P*(**y**|**f**)*P*(**f**|**X**, **θ**)*d***f**, and **θ** is a vector that collects the hyperparameters. To perform inference several model hyperparameters **θ** must be set. These include the covariance function parameter log(*s*^2^) (controlling the scale and bias) and the likelihood parameters {*b*_1_, log(Δ_2_), …,log(Δ_*R* − 1_), log(*σ*)}. Note, that each hyperparameter should be greater than zero except for *b*_1_, so to enforce positivity we optimise all other variables in the log domain. The normalisation factor *P*(**y**|**X**, **θ**) is known as the model evidence and is the metric used to learn the hyperparameters. To approximate the posterior distribution and model evidence we use the Laplace approximation at the maximum a posteriori (MAP) estimate (Williams and Barber, 1998). This approach was found to perform similarly to an expectation propagation approach across nine benchmark data sets (Chu and Ghahramani, 2005). Powell's method is used to maximise the evidence and hence, infer the optimal hyperparameters (Powell, 1964).

Having set the hyperparameters, we now want to make predictions about a test case x_*_ for which the target y_*_ is unknown. Under the Laplace approximation, the predictive distribution for the latent function can be written as a Gaussian $N\left(f\left(x_{*}\right);\mu_{*},\xi_{*}^{2}\right)$ where the predictive mean and variance can be written as

$$\mu_{*}=k_{*}^{T}K^{-1}\hat{f}\;\mathrm{and}\;\xi_{*}^{2}=k_{**}-k_{*}^{T}{\left(K+{\hat{\Lambda }}^{-1}\right)}^{-1}k_{*},$$

where k_*_ is the covariance between the test case and the training data, k_**_ is the variance of the test case, $\hat{f}$ is the MAP estimate of the latent function, $\hat{\Lambda }$ is a diagonal matrix whose *ii*-th entry is second derivative of the likelihood function training sample *i* with respect to *f*(**x**_*i*_).

The predictive distribution over the ordinal target y_*_ is

$$P\left(y_{*}|x_{*},X,y,\theta \right)=\Phi \left(\frac{b_{y_{*}}-\mu_{*}}{\sqrt{\sigma^{2}+\xi_{*}^{2}}}\right)-\Phi \left(\frac{b_{y_{*}-1}-\mu_{*}}{\sqrt{\sigma^{2}+\xi_{*}^{2}}}\right)\text{.}$$

This distribution is used to assign the test case to an ordinal scale using

$$\arg\max_{r\in R}P\left(y_{*}=r|x_{*},X,y,\theta \right)\text{.}$$

### Multi-class classification

Here we present two approaches for multi-class classification using Gaussian processes. The first involves forming pairwise binary classifiers and computing the test label for an unseen test case using error correcting codes while the second is an inherently multi-class approach which considers all classes simultaneously. Both of these approaches have been used to provide a comprehensive comparison of ORGP with multi-class classification.

#### Pairwise multi-class classification using Gaussian processes (PMCGPs)

This approach involves solving the multi-class classification problem using binary classifiers where all pairs of classes are compared to each other. Given a three class problem we can build three unique pairwise classifiers where each binary classifier votes for either of the two classes it was trained on to produce a 3-bit predicted code. In order to assign a label to a test case, error correcting codes were used whereby each class is assigned a unique codeword and its distance from the predicted code word is computed (Dietterich and Bakiri, 1995). The test case is assigned to the class with the minimum distance from its pre-defined code word. Here, binary classification was implemented using Gaussian process learning as described by Rasmussen and Williams (2006) and provides probabilistic measures of class membership. Therefore, probabilistic code words were used, see Table 1.

#### Multi-class classification using Gaussian processes (MCGP)

Gaussian process classification can be extended from the binary case using a multi-class analogue of the logistic function (the response function which maps the values of the latent function onto [0 1] to produce probabilities) — i.e. the softmax function (Williams and Barber, 1998). Here, we provide a brief summary of the mathematical concepts introduced by Williams and Barber for MCGP but refer the reader to Williams and Barber (1998) and Rasmussen and Williams (2006) for a more detailed description. In this case, an independent latent function is used to model the probability of each class.

Consider a vector of latent function values at all training points *N* and for all *R* classes

$$f^{\mathrm{MC}}=\left(f_{1}^{1},\ldots ,f_{N}^{1},f_{1}^{2},\ldots ,f_{N}^{2},\ldots ,f_{1}^{R},\ldots ,f_{N}^{R}\right)$$

where **f**^*MC*^ has length *RN*, i.e. each class has a corresponding set of *N* latent variables. As before, the prior over **f**^*MC*^ has the form $f^{\mathrm{MC}}\sim N\left(0,K\right)\text{.}$ Assuming that the latent variables for each class are uncorrelated, the covariance matrix ***K*** is a block diagonal in the matrices **K**_1_, …,**K**_*R*_. Each individual matrix *K*_*r*_ expresses the covariance of the latent function for each class *r* using an individual covariance hyperparameter *s*_*r*_^2^ to control the variance and bias so that,

$$K_{r}=\left(XX^{T}+1\right)/s_{r}^{2}\text{.}$$

Let *π*_*i*_^*r*^ denote the output of the softmax function at training sample *i*

$$p\left(y_{i}=r|f_{i}^{\mathrm{MC}}\right)=\pi_{i}^{r}=\frac{\exp\left(f_{i}^{r}\right)}{\sum_{d=1}^{R}\exp\left(f_{i}^{d}\right)}$$

where **f**_*i*_^*MC*^ is a vector of length *R* describing the latent function values across all classes for training sample *i*. This formulation enforces the constraint ∑_*r* = 1_^*R*^*π*_*i*_^*r*^ = 1.

As in the binary case and for ordinal regression we seek the maximum a posteriori value ${\hat{f}}^{\mathrm{MC}}$ of the posterior *P*(**f**^*MC*^|**y**, **X**, **θ**) which is achieved using Newton's method as described in Rasmussen and Williams (2006). The Laplace approximation is then used to compute the posterior. As in the ordinal regression approach we use Powell's method to optimise the hyperparameters of the covariance function. To compute the predictive distribution we apply the softmax function to the approximated posterior distribution of the latent function evaluated at the test point. For binary classification, class assignment is achieved by thresholding the predictive mean, however, for multi-class classification we need to take the variance of the mean into account, which is computed by a simple Monte Carlo procedure as in Rasmussen and Williams (2006).

### Metric regression

Metric regression was implemented using ridge regression (RR) which involves linear least square regression with Tikhonov regularisation (Bishop, 2006). Here, RR was implemented using the dual representation, i.e. learning is performed in the dimension of samples rather than features. It is therefore equivalent to the kernel ridge regression approach described for neuroimaging data by Chu et al. (2011) using the formulation,

$$\alpha ={\left(XX^{T}+\lambda I\right)}^{-1}\left(y-\bar{y}\right)$$

where *λ* controls the level of regularisation and $\bar{y}$ is the (scalar) mean across all training labels. To encode the ordinal relationship across the three classes the labels [1 2 3] are used, in keeping with the data labels used for the ORGP approach. Predictions are made using

$$y_{*}=k_{*}^{T}\alpha +\bar{y}$$

where y_*_ are the real-valued predictions. To force the metric predictions to define distinct three-class categories we use a somewhat ad-hoc approach which involves rounding the predictions to the nearest integer; if this integer is 1 or 2 or 3 then the class label is directly assigned whereas if the integer is 0 it is assigned to class 1 and if the integer is greater than 3 it is assigned to class three. We assessed the effect of the regularisation parameter across a range of values and we found that the accuracy was insensitive to the value of particular setting of *λ*, therefore in keeping with Chu et al. (2011) we set *λ* to 10*e*^− 5^.

### Multivariate whole brain maps

For the Gaussian process algorithms which consider all classes simultaneously (i.e. ORGP and MCGP but not PMCGP) multivariate maps were constructed to visualise the spatial pattern driving the regression or classification. For GP learning, this is achieved by visualising the MAP estimate of the weight vector to provide a spatial representation of the decision boundary. This is analogous to the weight vector used for mapping SVM discrimination (Marquand et al., 2010). For both MCGP and ORGP we can extract a vector **α**, which is analogous to the weight vector in the function-space view of GP learning (Rasmussen and Williams, 2006), whereby **α** = **K**^− 1^**f**.

To our knowledge, this is the first paper to introduce discriminative mapping for ordinal regression using Gaussian processes. For ORGP, the maps are constructed by computing the posterior expectation $\hat{w}$ of the weight vector in the weight-space

$$\hat{w}=\frac{1}{s^{2}}X^{T}\alpha \text{.}$$

For MCGP, the MAP expectation of the weights can be derived similarly for each class. Since multivariate techniques are sensitive to spatial correlation, and the performance of the classifier is based on the entire pattern rather than individual voxels, inference based on local regions should be avoided when interpreting these maps.

### Implementation of pattern recognition techniques

All algorithms were trained in a leave-one-out cross-validated manner, whereby data from all but one of the participants were used to train the model and the final (unseen) participant's data were used for testing.

As ordinal regression is used to predict the ordering of unseen data, we will present the results using familiar classification metrics (e.g. accuracy, confusion matrices) but also metrics that are designed to appropriately penalise the errors made when predicting rank (mean absolute error and Kendall's tau).

We use confusion matrices as visualisation tools. For confusion matrices, the rows represent the true class labels and the columns represent the labels predicted by the learning machine. The diagonal elements represent correctly classified test cases whereas the off-diagonal elements represent misclassifications. Thus, this matrix represents the performance of the learning machine on a per-class basis and also enables us to visualise the magnitude of the errors, i.e. errors between adjacent classes versus errors in more distal classes. The accuracy is calculated from this matrix by dividing the sum along the diagonal by the sum of all cells. The per class sensitivities are defined as the number of true positives divided by the sum of the number of true positives and false negatives. The positive predictive value (PPV) the probability of belonging to a particular class given that class membership was assigned by the algorithm. It is calculated as the number of true positives divided by the number of true positives and false positives.

Mean absolute error (MAE) is the average deviation of the predicted label from the true label whereby $\mathrm{MAE}=\frac{1}{N}\sum_{i=1}^{N}\left|y_{i}-\left({\left(y_{*}\right)}_{i}\right)\right|\text{,}$ where *N* is the total number of samples and (y_*_)_*i*_ is the predicted label for sample *i*. Note, for RR MAE is calculated from the real-valued predicted outputs to capture its behaviour in a fine-grained manner. The rank correlation coefficient between the predicted label and the true label was calculated using Kendall's tau statistic (Kendall, 1938).

In the context of probabilistic models, the theoretically optimal quantity for model comparison is the marginal likelihood, which provides an optimal trade-off between model fit and complexity under the assumptions of the model. However, to compare different models using the marginal likelihood it is necessary to also account for different numbers of hyperparameters by integrating them out or penalising models with a larger number of parameters. In GP models, the hyperparameters can only be integrated out using Markov chain Monte Carlo methods, which are relatively computationally demanding. Therefore, in the present work we adopt an alternative approach to compare the ordinal regression model to the explicitly multi-class model (MCGP). Specifically, we penalise the marginal likelihood by the number of parameters to construct both the Akaike information criterion (corrected for finite sample size) (Akaike, 1974) and the Bayesian information criterion (Schwarz, 1978). Both of these measures are widely used in neuroimaging and assess model fit while accounting for model complexity (in this case, the number of hyperparameters) which is important here as ORGP has an additional hyperparameter relative to MCGP. However, the Bayesian information criterion penalises complexity more strongly. Note that the MCGP is parameterised using three hyperparameters (one per class). However, the constraint that the class probabilities must sum to one introduces a redundancy such that the likelihood can be equivalently represented using two hyperparameters by fixing the latent function values for one of the classes (the reference class) to one. Therefore, to be conservative we penalise the MCGP likelihood by two (rather than three) parameters to compute the information criteria although we note that both approaches lead to the same conclusions.

All algorithms were implemented in MATLAB (The MathWorks, Natick, Massachusetts). For ORGP, custom likelihood and inference scripts were written for compatibility with the GPML toolbox (Rasmussen and Nickisch, 2010). MCGP was implemented as per the PRoNTo toolbox (Schrouff et al., in press). PMCGP was implemented using customised scripts also from the PRoNTo toolbox.

### Significance assessment

Statistical significance was assessed by testing if the accuracies achieved were greater than chance (33%) using permutation testing. The class labels were randomly permuted 1000 times and the learning algorithms (ORGP, PMCGP, MCGP and RR) were re-trained using these labels. The number of times the permuted accuracy was greater than the true accuracy was counted and divided by the number of permutations to produce a p-value. To assess the difference between the accuracy achieved for ORGP versus all other algorithms the permuted difference in accuracies was computed. The number of times the permuted difference was greater than the true difference was divided by the number of permutations to produce a p-value.

### Ketamine study

Sixteen healthy male participants (mean age 25.8 years; SD = 5.7; range 20–37) with no previous neurological or psychiatric illness or history of alcohol or drug abuse were recruited and completed four scanning sessions. All participants gave written informed consent. The study was approved by Wandsworth Research Ethics Committee (09/H0803/48). Further details for the participants and experimental procedures are available in Doyle et al. (2013). The design is briefly summarised below.

This randomised placebo-controlled, double-blind, partial crossover design involved screening and four scanning visits. For each scanning visit, participants received: placebo (ascorbic acid) and saline infusion, placebo and ketamine infusion, lamotrigine (300 mg) and ketamine infusion and risperidone (2 mg) and ketamine infusion. The oral drugs were administered in identical capsules approximately 3 h prior to ketamine infusion.

#### Image acquisition

Participants were scanned using a 3.0T General Electric Signa HDx scanner (GE Medical Systems, Milwaukee, WI, USA). A 15-minute eye-open resting-state BOLD phMRI scan was acquired using gradient-echo echo-planar imaging (EPI). 450 image volumes of 38 near-axial slices (3 mm thickness, interslice gap of 0.3 mm) were acquired per session (TE/TR = 30/2000 ms, flip angle (FA) = 75°, in-plane resolution = 3.3 mm, matrix size = 64 × 64, field of view = 21.1 cm × 21.1 cm). A higher resolution gradient echo scan was also acquired (43 3 mm-thick near-axial slices with 0.3 mm gap, TE/TR = 30/2000 ms, FA = 90°, in-plane resolution = 3.3 mm, matrix size = 128 × 128, field of view = 24 cm × 24 cm).

#### Image pre-processing and modelling

The data were pre-processed using SPM5 (www.fil.ion.ucl.ac.uk/spm). This involved slice-timing correction, realignment, co-registration to the high-resolution image, spatial normalisation to the SPM EPI template using parameters derived from non-linear normalisation of the high-resolution image, and spatial smoothing (8 mm FWHM Gaussian kernel). A high-pass filter with a cut-off of 1200 s (twice the post infusion phMRI scan duration) was applied to the data to minimise the influence of very low frequency noise.

First-level modelling was performed in a general linear model framework as described previously (De Simoni et al., 2013). The design matrix comprised four regressors: 1) a gamma variate function parameterised to capture the BOLD response to a ketamine infusion in an independent cohort (for further details see De Simoni et al. (2013)), 2) the first component of a singular value decomposition of the six head motion traces, 3) a regressor to capture variations in the shape of the phMRI regressor and 4) a linear drift term. The beta images from the contrast of the gamma variate regressor were used as inputs for the pattern recognition analysis. These images contained 178,898 in-brain voxels across all 16 subjects. Here, we will apply our algorithms to the data using two configurations:

1. class 1: placebo–saline session, class 2: lamotrigine–ketamine and class 3: placebo–ketamine
2. class 1: placebo–saline session, class 2: risperidone–ketamine and class 3: placebo–ketamine.

For brevity, we will refer to configuration 1 as the lamotrigine case and configuration 2 as the risperidone case. As mentioned in the Introduction, we cannot rank lamotrigine and risperidone in a meaningful, principled manner, hence we will apply the algorithms separately.

### Scopolamine

15 healthy male volunteers (mean age 24.9 years; SD = 6.6; range 19–43) participated in a three period Latin-square design study, and were administered oral placebo and saline (s.c.), placebo and scopolamine (0.2 mg s.c.; SCOP) or donepezil (5 mg p.o.) and scopolamine. The donepezil and placebo were administered in identical capsules. Whole brain rCBF was measured using pulsed-continuous ASL (pCASL). Participants passed a screening period as described for the ketamine study above and the study was approved by the Institute of Psychiatry and South London and Maudsley NHS Trust Research Ethics Committee (06/Q0706/52).

#### Image acquisition

Subjects rested in the scanner while a single whole brain cerebral blood flow map was acquired using a pulsed-continuous ASL sequence (Dai et al., 2008). In this technique, arterial blood is labelled using a long (1.5 s) train of Hanning-shaped radio frequency pulses of 500 μs duration and 500 μs inter-pulse gap. This method labels the longitudinal magnetisation of arterial blood by means of a flow-driven, adiabatic inversion in the presence of a net magnetic field gradient of 0.07 G cm^− 1^. After a post-labelling delay of 1.5 s, the images were acquired with a 3D spiral multi-short readout (TE 4 ms, TR 5500 ms, ETL 64). CBF maps (in standard physiological units (millilitres of blood per 100 g of tissue per minute)) were computed with a spatial resolution of 1 × 1 × 3 mm. Three pairs of tagged–untagged images were collected; which together with two reference volumes for flow quantification, required a total acquisition time of 6:08 min. A high-resolution, anatomical T2-weighted scan was also acquired. This scan was collected with a Fast-Spin echo protocol (echo train length 19, TE/TR of 54.48/4380 ms); field of view 24 cm, 72 near axial slices along the AC–PC axis with 2 mm thickness and zero gap, were collected. The final in-plane resolution of the image was 1 × 1 mm.

#### Image pre-processing and region of interest definition

Extra-cerebral signal from the T2 structural scan was removed using the brain extraction tool included in FSL (Smith, 2002) and the skull-stripped T2 image and its corresponding binary mask were co-registered to each pCASL image using SPM5. Then the brain mask derived from the T2 image was applied to each pCASL image and the resulting skull stripped images were then co-registered back to the original T2 image. Finally, the high resolution T2 image was used to compute SPM5 normalisation parameters necessary to warp the image to the T2 MNI template provided with SPM5 and the resulting parameters were applied to the co-registered pCASL images in addition to the T2 image. Following normalisation, each whole-brain pCASL image was spatially smoothed (8 mm isotropic Gaussian kernel). Since, global basal rCBF values are potentially different between participants, each image was then mean-centred within participants.

To date, the authors are aware of only two studies describing the effect of donepezil pre-treatment on scopolamine in healthy volunteers, both of which involve cognitive paradigms (Lenz et al., 2012; Snyder et al., 2005). Therefore, we use the literature on scopolamine administered in healthy volunteers to guide our choice of ROIs (Grasby et al., 1995; Sperling et al., 2002). Following scopolamine administration, Grasby et al. (1995) reported sites of altered rCBF (measured using positron emission tomography) in the occipital cortex, thalamus, precuneus and premotor area. In keeping with the work of Grasby et al. (1995) and studies in the primate (Asai et al., 2009; Yamamoto et al., 2011), we have selected the thalamus and occipital cortex as regions of interest. Additionally, we have selected the anterior cingulate cortex (ACC) which receives intense cholinergic innervation from a collection of cells in the basal forebrain (Mesulam, 1995) and therefore we hypothesized that rCBF in the ACC would be altered by scopolamine and its modulation with donepezil.

Here, we will apply our algorithms to the data using the configuration:

class 1: placebo–saline, class 2: donepezil–scopolamine and class 3: placebo–scopolamine

For brevity, we will refer this configuration as the donepezil case.

## Results

Ordinal regression (ORGP), multi-class classification (PMCGP and MCGP) and metric regression (RR) were applied to brain imaging data to provide predictions of class membership (probabilistic in the case of ORGP and MCGP). We compare the performance of these algorithms across pharmacological imaging data sets for which we expect an ordinal relationship to exist. For the BOLD data we use whole brain data from the placebo and ketamine sessions at the extremities with either lamotrigine or risperidone as the intermediate class. For the ASL data we explore the behaviour in three ROIs across the placebo and scopolamine session at the extremities with donepezil as the intermediate class.

### Ketamine Study

**Fig. 2:**
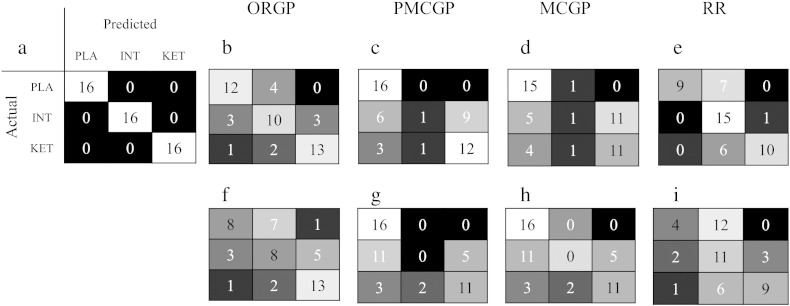
Confusion matrices using both lamotrigine (LAM) and risperidone (RIS) as the intermediate class for ORGP, PMCGP, MCGP and RR. The greyscale which is provided for visualisation with bright colouring in the diagonal and dark colouring off-diagonal indicates good performance. (a) Visualisation of the ideal confusion, (b) placebo–lamotrigine–ketamine for ORGP, (c) placebo–lamotrigine–ketamine for PMCGP, (d) placebo–lamotrigine–ketamine for RR, (e) placebo–risperidone–ketamine for ORGP, (f) placebo–risperidone–ketamine for PMCGP, (g) placebo–risperidone–ketamine for MCGP and (h) placebo–risperidone–ketamine for RR.

The performance of all techniques is displayed as confusion matrices in Fig. 2 and the accuracy, mean absolute error (MAE), Kendall's tau, model evidence and information criteria are reported in Table 2 (note that the chance level for a three-class problem is 33.3%). For ORGP, considering lamotrigine as the intermediate class resulted in a mean classification accuracy across all three classes of 72.9% with sensitivities (PPV) of 5% (75%), 62.5% (62.5%) and 81.3% (81.3%) for placebo, lamotrigine and ketamine, respectively. In Fig. 2, we can see that for the placebo class, four scans were misclassified. However, the errors were confined to the adjacent class — lamotrigine. For ketamine, three scans were misclassified two of which occurred for the adjacent class with only one assigned to the more distant placebo class. The misclassifications for lamotrigine were distributed evenly across the adjacent classes. However, the misclassification errors tended to occur for the classes at the extremities with seven of placebo scans and 6 of the ketamine scans misclassified. ORGP significantly outperformed all other techniques considering the MAE (p = 0.035, 0.014, 0.04, for PMCGP, MCGP and RR, respectively). Additionally, PMCGP and MCGP but not RR were significantly less accurate than ORGP (p = 0.03 and p = 0.04, respectively, by permutation test) in discriminating the groups with overall accuracies of 60.4%, 56.3% and 70.8%, respectively. Crucially, for both multi-class approaches only one of the scans belonging to the intermediate class was correctly labelled (Fig. 2). Similarly for RR, the misclassifications were less evenly distributed amongst classes than for ORGP.

Considering risperidone as the intermediate class, the mean accuracy across the three classes was 60.4% for ORGP which is similar to the mean accuracy for both multi-class approaches which was 56.3%. Additionally, similar levels of performance were observed for MAE and Kendall's tau, Table 2. However, the distribution of the errors across the classes sets the techniques apart: for both multi-class approaches none of the risperidone scans are labelled correctly, see Fig. 2. Unlike the multi-class schemes RR achieved correctly identified 11 out of 16 of the risperidone scans however, the true positive for the placebo class was much reduced with ORGP found to significantly outperform RR in terms of accuracy and MAE, (p = 0.04 and p = 0.001, by permutation).

**Fig. 3:**
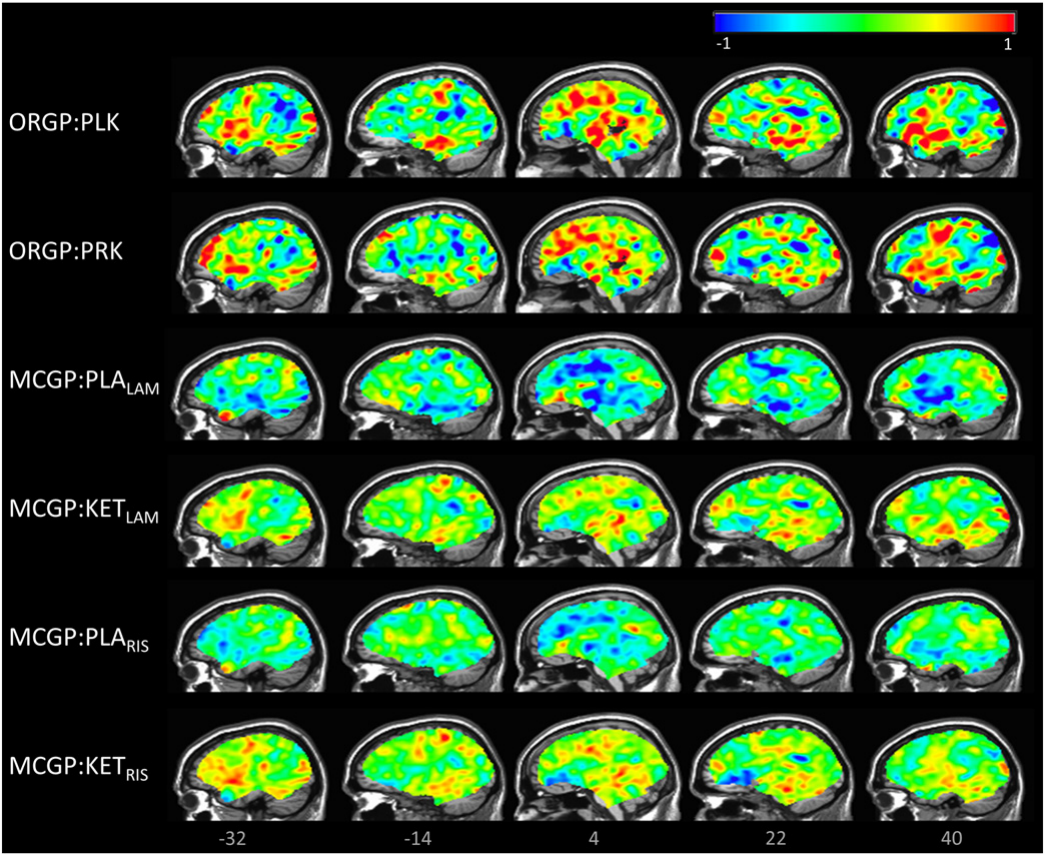
Multivariate maps extracted from both ORGP and MCGP. For ORGP, a single weight map is produced whereas, for MCGP a weight map can be computed per class. Only weight maps with significant classification accuracy were computed. For visualisation, each map is scaled so that its maximum (absolute) intensity is 1. ORGP:PLK — ordinal regression weight vector for all three classes. ORGP:PRK — ordinal regression weight vector for all three classes considering risperidone as the intermediate class. MCGP:PLA_LAM_ — multi-class classification weight vector for the placebo class considering lamotrigine as the intermediate class. MCGP:KET_LAM_ — multi-class classification weight vector for the ketamine class considering lamotrigine as the intermediate class. MCGP:PLA_RIS_ — multi-class classification weight vector for the placebo class considering risperidone as the intermediate class. MCGP:KET_RIS_ — multi-class classification weight vector for the ketamine class considering risperidone as the intermediate class.

For ORGP and MCGP, the Akaike and Bayesian information criteria were lower for the ordinal regression models in both cases, indicating that these ordinal models were favoured over the multi-class approach. Whole brain maps for the lamotrigine and risperidone cases can be observed in Fig. 3. For ORGP, this map visualises the projection of the data along the function space weight vector (*α*) moving from the placebo state right through the ketamine state. Qualitatively, the maps generated by ORGP for both lamotrigine and risperidone are similar (see Fig. 3). For MCGP, none of the lamotrigine/risperidone classes were correctly classified, so the weight maps for these classes are not meaningful. Therefore, we only present the maps for the placebo and ketamine classes.

### Scopolamine study

Here, we focus on two cortical regions (anterior cingulate cortex and occipital lobe) and a sub-cortical region (thalamus). As before, the performance of all four techniques is displayed using confusion matrices in Figure S1 and using a collection of metrics in Table 3. For the ACC ORGP was found to significantly outperform all other techniques in terms of classification accuracy and also MAE. Notably, for the anterior cingulate, ORGP achieves over a 20% increase in classification performance relative to all other approaches. For the thalamus, ORGP outperformed PMCGP in terms of accuracy (p = 0.002) and both PMCGP and RR in terms of MAE (p = 0.007 and p = 0.039, respectively). Conversely, similar performance was observed across all techniques for the occipital lobe. A potential explanation for this result can be observed from Figure S2 which displays the average pCASL signal for each ROI. For both the ACC and thalamus, in a qualitative sense, we observe a decreasing rCBF ordinal response from placebo to scopolamine with increased overlap between classes observed for the ACC. However, the group-level rCBF for the occipital lobe donepezil pre-treatment appears to have reversed the decrease in rCBF resulting in no visually obvious ordinal response.

## Discussion

We have presented a multivariate ordinal regression methodology for neuroimaging data. We have demonstrated the efficacy of this novel analysis method in the context of two pharmacological imaging studies. Amongst the approaches evaluated, ORGP was the only approach that performed well in all comparisons and produced higher or equivalent accuracy and mean absolute error to the optimal approach in every comparison. For the ketamine study, multiclass classification, ordinal regression and metric regression were able to classify the ordinal groups with accuracies significantly above chance. The ORGP approach presented here considers all classes simultaneously in a Bayesian framework affording probabilistic predictions and an elegant approach to model selection. Here, we draw particular comparison with an algorithmically similar multiclass approach: ordinal regression significantly outperformed the inherently multi-class approach for lamotrigine (72.9% vs. 56.3%, p = 0.014) while a similar performance across both techniques was observed for risperidone (60.4% vs. 56.3%). Crucially, for the intermediate classes, the true positive rates obtained for ORGP were 62.5% and 50% for lamotrigine and risperidone (chance level is 33.3%) but in the case of MCGP they were reduced to 6.3% and 0%, respectively. Moreover, both the Akaike and Bayesian information criteria were lowest for the ordinal regression models having conservatively penalised for the number of hyperparameters. Metric regression (ridge regression) was found to perform similarly to ordinal regression for lamotrigine whereas for risperidone the accuracy for ORGP was significantly higher; recalling that for RR the approach to achieve categorical labels is ad-hoc. Additionally, for the scopolamine data set ORGP significantly outperformed all other approaches for the anterior cingulate cortex. Note, we also implemented Gaussian process regression, however, the method did not perform in a stable manner which we attribute to the highly non-Gaussian nature of the encoded labels (i.e. a high degree of model mis-specification).

While our exemplar analysis is based on psychopharmacological data, many neuroimaging studies involve ordinal scales, for which we cannot explicitly quantify the distance between classes. As mentioned in the Introduction, this might include complexity of a cognitive task, clinical status, visual analogue scales (subjective states), pain intensity, disease progression/transition and genetic dosage to name some examples. To date, we are aware of only two other neuroimaging studies that investigate the use of ordinal schemes for their data. Fan and ADNI (2011) applied an ordinal ranking approach to the ADNI data set with the hypothesis that an ordinal approach is more appropriate for the automatic identification of the following four class labels — healthy controls, mild cognitive impairment non-converter, mild cognitive impairment converter and Alzheimer's disease. Fan proposed a method that divides the ordinal regression problem into a series of binary ‘larger than’ classifiers and compared it to a multi-class classifier formulated using one-against-one binary pairs of classifiers. While the overall accuracies for the ordinal and multi-class approaches did not differ, the distribution of the errors was evenly spread across all four classes for the ordinal approach but skewed towards both MCI classes in the case of the multi-class classifier. This trend is similar to the results reported here whereby for some cases the accuracy for the intermediate classes shows a marked improvement on using an ordinal approach. Pedregosa et al. (2012) investigated the use of presenting all possible class-wise pairs of images to a binary classifier in order to encode information about the ordinal relationship between the class labels. This approach was investigated using fMRI data acquired while subjects were listening to sentences with five increasing levels of complexity. The authors conclude that the analysis revealed a non-linear relationship between the BOLD signal response and the level of complexity. However, further validation is warranted to fully understand the potential of their technique. Moreover, Pedregosa's approach requires that test cases for each possible class be available in order to decode the ranking label.

Here, we have presented a novel whole brain approach to ordinal regression that considers all classes simultaneously, which is distinct from previous studies to date (Fan and ADNI, 2011; Pedregosa et al., 2012). The implementation provides an explicit probabilistic model, which is formulated in a Gaussian process framework that provides flexibility in quantifying the pair wise similarity between *samples*, e.g. linear and non-linear kernels. Kernel methods such as Gaussian process methods are computationally advantageous when the input features exist in a high dimensional space, as is the case for neuroimaging data, because they enable us to work in dimensions of samples rather than features. While not explored here, kernel methods also provide an elegant method for the combination of multimodal data (Gonen and Alpaydin, 2011). The application of learning algorithms in a neuroimaging context is often an ill-posed problem, as the number of features (voxels) generally greatly outnumbers the number of samples (scans). To help alleviate the possibility of overfitting, we have employed algorithms that incorporate regularisation examples which include penalising complexity and reducing the data to a subset of relevant features. Achieving an optimal balance between fitting the training data and penalising the magnitude of the coefficients is paramount (Ashburner and Kloppel, 2011). The Bayesian framework used here provides an elegant solution to regularisation by integrating out the parameters of the model, instead of estimating only the hyperparameters. Moreover, we have restricted the hypothesis space by choosing a linear classifier.

The multivariate whole brain maps computed for ORGP displayed patterns similar to a placebo vs. ketamine (visual inspection) response, as observed using binary Gaussian process classification (Doyle et al., 2013). Additionally, the MCGP maps extracted for the ketamine class are also highly similar to ORGP maps. This may indicate that a similar set of features are highly weighted in the context of multi-class/binary classification and ordinal regression across the continuum. Therefore, while both techniques weight the same set of features in a similar fashion, ordinal regression affords increased sensitivity over MCGP by explicitly modelling the ranking of the classes as evidenced by its superior performance across a range of metrics — accuracy, mean absolute error and information criteria. One of the data sets employed here involves the administration of the compound ketamine, which is known to cause a widespread but selective activation across brain regions (De Simoni et al., 2013). Conversely, for the scopolamine data we anticipated that altered rCBF and its modulation with donepezil may only occur in areas rich in M_2_ receptors and therefore we chose regions of interest appropriately. However, when limited prior knowledge is available with regard to the spatial distribution of the response we suggest applying ordinal regression locally using a searchlight approach (Kriegeskorte et al., 2006) to provide the whole brain accuracy or mean absolute error maps. Given the local nature of their computation, local inference could be applied to these maps to establish where the imaging data is most related to the ordinal target. Another interesting avenue for future work would be to develop an approach to enforce sparsity over the voxel coefficients, which may be useful to help localise discriminating brain regions.

In summary, we have presented an application of probabilistic multivariate ordinal regression to neuroimaging data. To rigorously validate this proposal we would need to apply this technique to additional data sets that focus on varied applications such as disease progression and cognitive decoding. However, using pharmacological imaging data as an exemplar, we have confirmed that in some cases presented here ordinal regression outperforms multi-class classification schemes as well as metric regression, particularly for the intermediate classes. This suggests that the methodology presented here may afford increased sensitivity for a wide range of neuroimaging applications.

## Figures and Tables

**Table 1:** Probabilistic code words for each class. C1 — class 1, C2 — class 2 and C3 — class 3. ‘0’ implies that the output from the binary classifier is expected to be the first class listed. Similarly, ‘1’ implies the second class listed. ‘0.5’ implies that the classifier is neutral, for example testing an instance from class 3 on the classifier trained on class 1 and class 2.

	Binary classifier pairs		
	C1 × C2	C1 × C3	C2 × C3
C1	0	0	0.5
C2	1	0.5	0
C3	0.5	1	1

**Table 2:** Performance metrics for ordinal regression (ORGP) and pairwise multi-class classification (MCGP), multi-class classification (MCGP) and ridge regression (RR). Model evidence is quantified using the negative marginal log likelihood computed using the entire data set. LAM: lamotrigine, RIS: risperidone, MAE: mean absolute error, AIC: Akaike information criterion, and BIC: Bayesian information criterion.

Performance metric	LAM (ORGP)	LAM (PMCGP)	LAM (MCGP)	LAM (RR)	RIS (ORGP)	RIS (PMCGP)	RIS (MCGP)	RIS (RR)
Accuracy	72.9%⁎†‡	60.4%⁎	56.3%⁎	70.8%⁎	60.4%⁎§	56.3%⁎	56.3%⁎	50.0%⁎
MAE	0.29†‡§	0.46	0.52	0.40	0.44§	0.50	0.50	0.61
Kendall's tau	0.70	0.61	0.53	0.71	0.57	0.61	0.61	0.46
Model evidence	39.0	–	44.6	–	40.4		45.6	
AIC	86.9	–	93.5	–	89.7		95.5	
BIC	93.5	–	96.9	–	96.3		98.9	

⁎ Accuracy > chance (33%) p < 0.05.

† ORGP outperforms PMCGP p < 0.05.

‡ ORGP outperforms MCGP p < 0.05.

§ ORGP outperforms RR p < 0.05.

**Table 3:** Performance metrics for ordinal regression (ORGP) and pairwise multi-class classification (MCGP), multi-class classification (MCGP) and ridge regression (RR). Model evidence is quantified using the negative marginal log likelihood computed using the entire data set. MAE — mean absolute error, AIC: Akaike information criterion, BIC: Bayesian information criterion, and DON: donepezil.

	Performance Metric	DON (ORGP)	DON (PMCGP)	DON (MCGP)	DON (RR)
Anterior cingulate	Accuracy	73.3%⁎†‡§	40.0%⁎	51.1%⁎	42.4%⁎
MAE	0.29†‡§	0.73	0.51	0.69
Kendall's tau	0.70	0.21	0.53	0.32
Model evidence	24.8	–	38.8	–
AIC	58.6	–	86.6	–
BIC	64.8	–	92.8	–
Occipital lobe	Accuracy	64.4%⁎	64.4%⁎	60.0%⁎	64.4%⁎
MAE	0.378	0.40	0.46	0.38
Kendall's tau	0.63	0.59	0.52	0.60
Model evidence	27.7	–	35.9	–
AIC	64.4	–	80.8	–
BIC	70.6	–	87.0	–
Thalamus	Accuracy	80.0%⁎†	60.0%⁎	68.9%⁎	66.7%⁎
MAE	0.20†§	0.42	0.31	0.36
Kendall's tau	0.81	0.60	0.72	0.66
Model evidence	20.8	–	29.8	–
AIC	50.6	–	68.6	–
BIC	56.8	–	74.8	–

⁎ Accuracy > chance (33%) p < 0.05.

† ORGP outperforms PMCGP p < 0.05.

‡ ORGP outperforms MCGP p < 0.05.

§ ORGP outperforms RR p < 0.05.

## Appendix A Supplementary data

Supplementary material 1.

Supplementary material 2 Ordinal Likelihoods.

## Footnotes

2 A MATLAB implementation of this method is available on request from the corresponding author.
