# Supplementary material for: Multivariate decoding of brain images using ordinal regression
Source: Neuroimage. 2013 Nov 1;81:347–57. doi: 10.1016/j.neuroimage.2013.05.036 (PMC4068378; doi:10.1016/j.neuroimage.2013.05.036)
Supplement: Supplementary file 1 — Supplementary material 1. [file mmc1.docx]

Supplementary Material for Multivariate decoding of brain images using ordinal regression by **O.M. Doyle, J. Ashburner, F.O. Zelaya, S.C.R. Williams, M.A. Mehta and A.F. Marquand.**

| 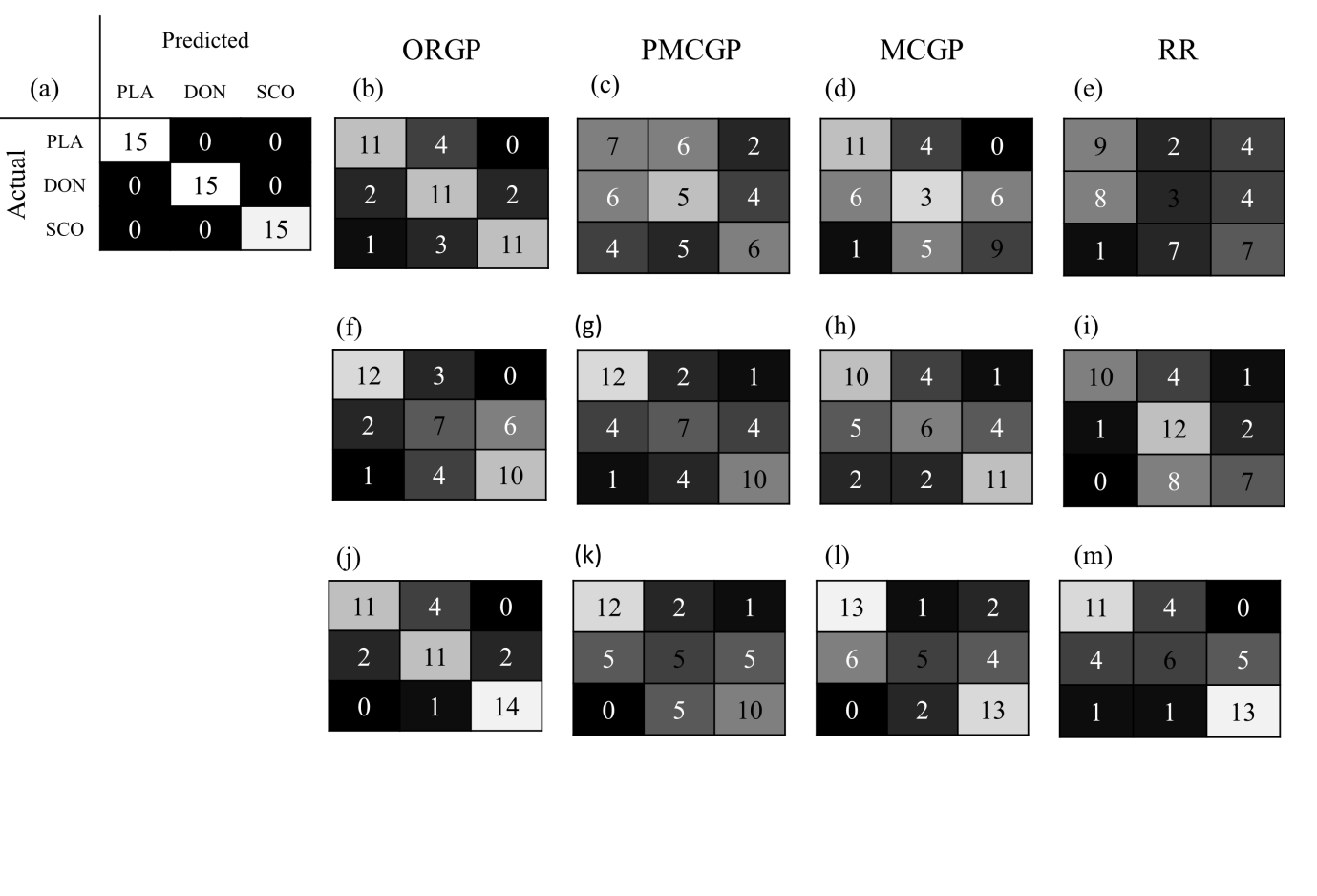 |
| --- |
| Figure S1: Confusion matrices for the scopolamine dataset with DON (donepezil) as intermediate class for ORGP, PMCGP, MCGP and RR. The grayscale is provided for visualisation with bright colouring in the diagonal and dark colouring off-diagonal indicate good performance. (a) Visualisation of the ideal confusion matrix, (b) ORGP for ACC, (c) PMCGP for ACC, (d) MCGP for ACC, (e) RR for ACC, (f) ORGP for occipital lobe, (g) PMCGP for occipital lobe, (h) MCGP for occipital lobe, (i) RR for occipital lobe, (j) ORGP for thalamus, (k) PMCGP for thalamus, (l) MCGP for thalamus and (m) RR for thalamus. |

| 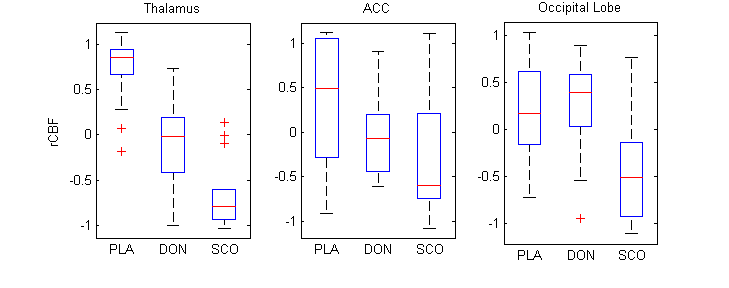 |
| --- |
| Figure S2: Group average regional cerebral blood flow values (rCBF) in normalised within subjects across three brain regions. |
